# Effect of Particle Size Control of Wheat Bran via Different Milling Techniques on the Structural and Physicochemical Properties of Arabinoxylan

**DOI:** 10.3390/molecules31091450

**Published:** 2026-04-27

**Authors:** Jeonghan Moon, Meera Kweon

**Affiliations:** 1 Department of Food Science and Nutrition, Pusan National University, Busan 46241, Republic of Korea; zzx276@pusan.ac.kr; 2 Kimchi Research Institute, Pusan National University, Busan 46241, Republic of Korea

**Keywords:** wheat bran, particle size reduction, milling methods, arabinoxylan, extraction yield

## Abstract

This study investigated how milling methods impact the extraction yield, structural features, and physicochemical properties of arabinoxylan (AX) isolated from wheat bran. Bran from three wheat cultivars (Goso, Hojoong, and Joongmo) was milled using an ultracentrifugal, mortar, or ball mill to generate fractions with different particle sizes. AX was extracted from each fraction and analyzed for yield, monosaccharide composition, arabinose-to-xylose (A/X) ratio, ferulic acid content, substitution patterns, and antioxidant-related indices. Ball milling produced the smallest particles and the highest AX yields, accompanied by increased ferulic acid release. NMR analysis indicated that ball milling reduced disubstituted xylose residues, suggesting partial disruption of highly substituted regions within the AX backbone. The A/X ratio varied by wheat type and milling method (0.44–0.60). Xylose and arabinose were the predominant monosaccharides, whereas residual glucose indicated incomplete starch hydrolysis. Ball milling also notably increased total phenolic content and ABTS radical scavenging activity, highlighting its role in releasing bioactive phenolic compounds. Overall, increased milling intensity improved AX extractability and enhanced the functional potential of wheat bran as a source of dietary fiber and antioxidant-active phytochemicals.

## 1. Introduction

Wheat bran is primarily produced as a byproduct during the commercial milling of wheat into flour. It is a rich source of dietary fiber—comprising approximately 50%—with non-starch polysaccharides as the major constituents. Among these, arabinoxylan (AX) accounts for ~70%, followed by cellulose (~24%), and β-glucan (~6%) [1,2]. AX is the principal component of cell walls in the endosperm and aleurone layers of cereal grains [3] and is present in whole wheat at levels ranging from 6.69% to 7.93%. Total AX (TAX) and water-extractable AX (WE-AX) contents are influenced by both genetic factors (e.g., variety) and environmental factors (e.g., agronomic conditions and climate), with a more pronounced genetic effect on WE-AX [4,5]. Structurally, AX consists of a xylose backbone substituted with arabinose residues at the C-2 and/or C-3 positions, whereas ferulic acid esterification occurs at the C-5 position of arabinose, facilitating covalent cross-linking with other molecules [6]. The substitution patterns of arabinose can be classified as unsubstituted, monosubstituted, or disubstituted [6,7]. The structural characteristics of AX, including molecular weight, arabinose-to-xylose (A/X) ratio, and degree of substitution, are influenced by grain source, cultivar, and environmental factors; these, in turn, affect physicochemical properties such as solubility and viscosity [8,9]. AX is commonly classified as water-extractable (WE) or water-unextractable (WU). Wheat bran AX is mainly water-unextractable [6,10,11], and differs from WE-AX in physical and chemical properties, including water absorption [11]. WU-AX has a higher molecular weight (approximately 200,000–3,000,000) and a higher arabinose substitution level and A/X ratio than WE-AX. However, its lower solubility results from the aggregation of large unsubstituted xylose (uXyl) regions, stabilized by hydrogen bonding and the formation of dehydrodiferulic bridges [4,6,11]. Regarding hydration, WU-AX increases water-holding capacity and moisture retention, while WE-AX increases viscosity. The health benefits of AX are mainly attributable to its fiber effects and fermentation by the gut microbiota, which functions as prebiotic by producing short-chain fatty acids and supporting gut health. AX also contributes to regulate glycolipid metabolism, immunomodulatory and anti-tumor activity and antioxidant properties [12].

To expand the applications of AX, efficient and cost-effective extraction techniques are essential to improve the yield and purity of AX due to its complex cell wall matrix. Pretreatment methods include milling, ultrasound, extrusion, and microwave processing [13,14,15,16]. Among these, milling is widely employed to enhance AX extractability by increasing the accessibility of cell wall constituents that are physically trapped in intact tissues. Reducing bran particle size increases cell wall rupture, surface area, and water penetration, thereby improving access for solvents and enzymes [17]. Demuth et al. [13] reported that milling wheat bran to a particle size of <0.5 mm significantly increased WE-AX yield and decreased both the A/X ratio and the average molar mass of WE-AX (from 403 kDa to 134 kDa) compared with un-milled wheat bran. The high energy input during milling may disrupt cell wall structure and break bonds within AX molecules, thereby increasing AX water solubility. To prepare small-scale batches of wheat bran with reduced particle size, numerous laboratory mills can be used. An ultra-centrifugal mill was demonstrated to be a useful tool for controlling particle size by combining high-speed centrifugal force and rapid rotor movement, resulting in shearing, extrusion, and rubbing actions to grind grains between the high-speed rotating rotor and ring sieves [18,19,20]. A mortar mill is another milling technique that consists of a mortar and a pestle: it relies on pressure and friction between the materials and the rotating pestle and generates relatively low heat due to its gentle grinding action. This minimizes the thermal degradation of heat-sensitive constituents and helps preserve the nutritional and functional integrity of the materials [21,22]. In addition, a ball mill pulverizes materials through direct collisions between the sample and grinding balls inside a jar, along with centrifugal force, effectively producing very fine particle sizes [23].

Regarding the effects of branny material particle size on AX extractability, ball milling of Psyllium (*Plantago ovata* Forsk) husk reduced the particle size from 160.9 μm to less than 6 μm, thereby increasing AX extractability from 13.2% to 98.4% [24]. Similarly, prolonged ball milling of wheat bran increased the A/X ratio and decreased the AX molecular weight to 32.4 kDa after 24 h and 14.6 kDa after 120 h [25]. Previous studies have examined the characteristics and molecular properties of AX extracted via specific milling techniques or from different wheat cultivars; however, limited information is available on how particle size reduction achieved by distinct milling techniques across different wheat cultivars influences AX extractability and composition [12,26,27]. Paesani et al. [28] reported the difference in the molecular size of soluble AX between hard and soft wheats, resulting in a significantly higher prebiotic effect for hard wheats. Therefore, we hypothesize that AX extractability and structural characteristics from hard and soft wheat bran are influenced by the extent and nature of bran particle size reduction induced by different milling techniques.

In this study, AX was extracted from the wheat bran of three cultivars—Goso (GS), Hojoong (HJ), and Joongmo (JM)—each developed for different end-use applications (cookies and crackers, noodles, and bread, respectively), although Korean cultivars are not classified as soft, semi-hard, or hard wheat. The brans were subjected to various milling techniques, resulting in unique particle sizes. Subsequently, extraction efficiency, physicochemical attributes (including functional properties), and structural features of AX—including substitution patterns, A/X ratio, and monosaccharide composition—were systematically evaluated.

## 2. Results and Discussion

### 2.1. Particle Size Distribution

**Figure 1:**
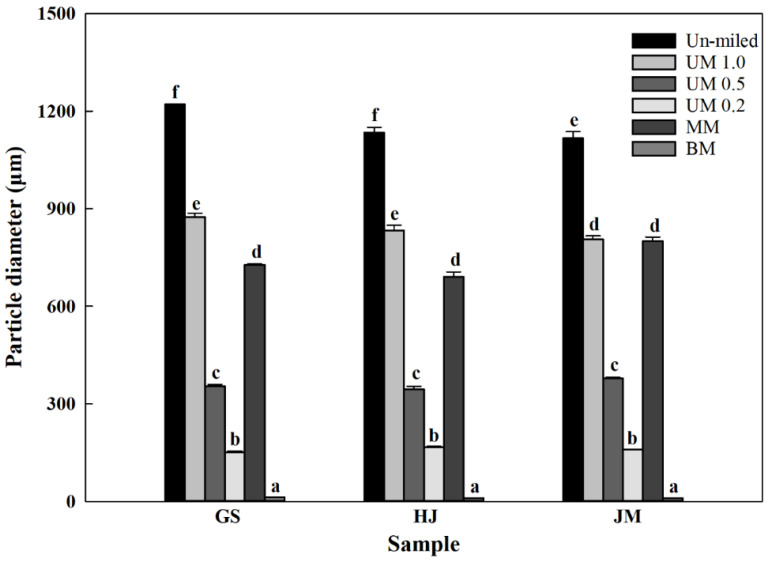
Particle size (D_50_) of wheat bran. GS: Goso, HJ: Hojoong, JM: Joongmo, UM 1.0, UM 0.5, and UM 0.2: ultracentrifugal mill with 1.0 mm, 0.5 mm, and 0.2 mm sieves, respectively; MM: mortar mill, BM: ball mill. Different letters above the bars indicate significant difference at *p* < 0.05 according to ANOVA with Tukey’s HSD test.

All milled bran samples exhibited smaller particle sizes (Figure 1) and higher span values (Table 1) than those of un-milled bran, confirming that milling effectively reduced the particle size. Among the milled samples, BM bran had the smallest particle size, whereas UM bran milled with a 1.0 mm sieve exhibited the largest particle size.

In contrast, the MM produced particle sizes of 1542, 1464, and 1621 μm, which decreased only slightly and remained comparable to those of UM 1.0 bran. This suggests that mortar milling has a limited efficiency, even with a longer milling duration. The heterogeneous, fiber-rich structure of wheat bran provides high flexibility [29], making it resistant to size reduction under the pressure and frictional heat generated between the rotating pestle and bran particles during mortar milling [30].

Meanwhile, BM produced significantly smaller particles (45, 29, and 30 μm), representing a remarkable reduction compared with UM- and MM-milled samples. Moreover, a peak in the 1.0 μm region (particle size distribution graph not shown) was observed only in BM bran, indicating that ball milling can generate ultrafine particles. Although ball milling typically requires a longer processing time, it is highly effective in producing fine particle sizes [23]. Milling is known to enhance the AX extraction yield by increasing the accessibility of cell walls to enzymes and chemicals during extraction [17].

In summary, the particle size of milled bran varies with milling technique and conditions. Among the samples, BM bran had the highest proportion of fine particles and is expected to exhibit greater AX extraction efficiency and a higher intracellular content.

### 2.2. Chemical Composition of Bran

The initial moisture contents of GS, HJ, and JM brans were 12.9%, 12.2%, and 11.8%, respectively (Table 2). Milling reduced moisture content, with a more pronounced decrease in UM 0.2 and BM bran. This was attributed to the frictional heat generated by the mechanical force and prolonged residence time within the milling chamber [31,32]. The recorded temperatures of the bran during milling were 25 °C for UM, 31 °C for MM, and 44 °C for BM.

The ash content ranged from 3.9% to 4.2% for GS, 3.6% to 3.8% for HJ, and 5.7% to 6.0% for JM, indicating that particle size reduction had no significant effect. The observed variation was primarily attributed to cultivar differences rather than to the milling treatment.

The damaged starch content of un-milled bran was 1.0%, 0.9%, and 1.2% for GS, HJ, and JM, respectively. After milling, starch damage increased markedly in MM and BM bran. Starch damage is greatly affected by the milling method’s mechanical characteristics [33] as starch granules undergo structural disruption through shear, impact, friction, and compression, resulting in particle fragmentation. In UM bran, starch damage increased only slightly or remained similar with decreasing particle size, likely due to the shorter blade contact time and brief milling duration [20]. Conversely, MM and BM bran, characterized by prolonged contact time and extended total milling duration, exhibited markedly higher starch damage [34]. When using wheat bran as an ingredient in baked products, controlling the level of damaged starch may be important because it can have either a negative or positive effect on processing and product quality. In bread baking, a moderate level of damaged starch (e.g., a falling number of 250 s) is desirable because it supports yeast fermentation during dough proofing; however, excessive damaged starch can result in bread with a lower loaf volume, a darker crust and a firmer crumb [35].

Within each cultivar, the total starch content followed the order: GS > HJ > JM. This trend remained relatively consistent across particle sizes, suggesting that milling had minimal impact on the intrinsic starch content. Wang et al. [36] similarly reported that total starch content is more strongly determined by cultivar type than by milling method.

Overall, milling influenced the chemical composition of wheat bran, with BM bran showing the lowest moisture content and the highest level of starch damage, likely due to longer milling time and higher energy input, although these parameters were not directly measured in this study. However, AX isolation from wheat bran commonly requires a destarching step using amylase and amyloglucosidase. Because damaged starch is more readily digested by these enzymes than native starch [37], starch removal is expected to be more efficient in BM bran than in other bran samples. This may be an additional benefit of BM for isolating AX from bran.

### 2.3. TAX and WE-AX Contents in Bran

Particle size reduction through milling increased the TAX (the sum of WU-AX and WE-AX) and WE-AX contents (Table 3). The BM bran exhibited the highest TAX levels, corresponding to the finest particle size. A similar trend was observed for WE-AX, where BM bran showed the highest values (1.79%, 2.02%, and 1.75%). The enhanced extraction of AX was likely due to the larger surface area and improved solvent accessibility provided by finer particles [38]. Across wheat cultivars, HJ, which had the finest particles based on D_90_, showed higher TAX and WE-AX contents than GS and JM.

Van Craeyveld et al. [24] also reported that prolonged ball milling of psyllium seed husks increased AX extractability from 15.7% to 98.4% when the milling time was extended from 2 to 168 h. Among the un-milled bran samples, TAX content was the highest in JM, followed by HJ and GS. Although the relative increase in TAX by the different milling techniques was not consistent across cultivars, particle size reduction resulted in an overall increase in TAX content. In contrast, the effect of milling on the WE-AX content was less pronounced and, in some cases, not significant compared with its impact on TAX. This difference may be explained by the composition and extractability of WU-AX and WE-AX in wheat bran. WU-AX is the predominant form in wheat bran, whereas WE-AX is present at lower levels. WE-AX is typically extracted first by water; in contrast, WU-AX is released-from the remaining wheat bran through enzymatic and chemical hydrolysis because AX is covalently bound within the cell wall matrix [39].

These findings suggest that particle size reduction via milling plays an important role in enhancing TAX extraction. Notably, ball milling is the most efficient technique for producing bran with the highest TAX and WE-AX contents.

### 2.4. Ferulic Acid Levels and Extraction Efficiency of AX from Wheat Bran

The AX yield from wheat bran samples ranged from 5.0% to 8.9% for GS, 4.9% to 11.2% for HJ, and 4.2% to 6.0% for JM (Table 4). Although a clear pattern was not observed in AX yield across the milling treatments, ball milling produced notably higher yields: 8.9% for GS, 11.2% for HJ, and 6.0% for JM. Niemi et al. [40] reported similar findings, showing that ball milling of brewer’s spent grain using three dry milling processes was an efficient approach for improving AX extractability. Among the cultivars, JM showed a lower extraction yield (4.2–6.0%) than those of GS and HJ. In addition, the AX yields from milled bran were comparable to, or lower than, those from un-milled bran. This reduction may be attributed to WE-AX loss during the extraction process, particularly after enzyme denaturation and supernatant removal, suggesting that milled bran samples are more prone to WE-AX loss than un-milled controls, contributing to fluctuations in AX levels.

The protein content of extracted AX ranged from 2.8% to 6.2% in GS, 4.0% to 6.8% in HJ, and 5.4% to 8.7% in JM. Although protease treatment during extraction reduced protein levels, considerable amounts remained within the AX fractions.

As a bioactive component in AX, the ferulic acid content ranged from 2.6 to 6.3 μg/mg in GS, 4.1 to 7.9 μg/mg in HJ, and 2.5 to 7.2 μg/mg in JM, with HJ showing the highest levels among the three cultivars. Ferulic acid is the predominant phenolic compound in wheat and is recognized for its strong antioxidant activity, contributing to the reduction in risks associated with cancer and diabetes and supporting overall health maintenance [41,42].

As particle size decreases during milling, BM produces the finest particles and, correspondingly, the highest AX yield and ferulic acid content.

### 2.5. Structural Properties of AX

**Figure 2:**
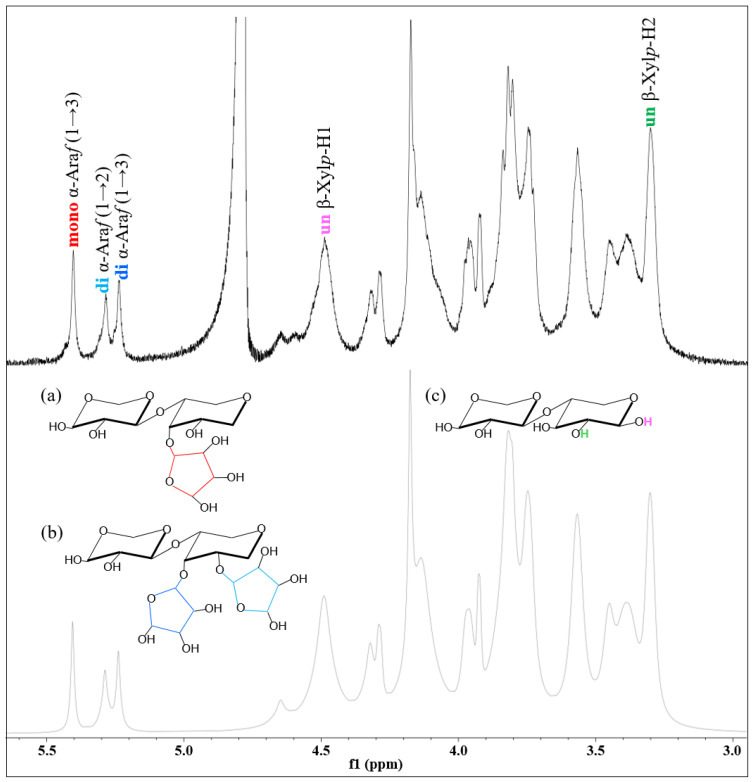
^1^H NMR spectra of arabinoxylan (AX) fractions correlate with substitution patterns of arabinofuranosyl (Ara*f*) and xylopyranosyl (Xyl*p*) residues. (**a**) Monosubstituted, (**b**) disubstituted, (**c**) unsubstituted.

The ^1^H NMR spectra revealed signals corresponding to the anomeric protons of Ara*f* units at δ 5.23, 5.28, and 5.40 ppm and those of Xyl*p* residues at δ 3.32 and 4.49 ppm [43]. The signal at δ 5.38 ppm was attributed to the single proton of Ara*f* (1→3) linkages, corresponding to monosubstitution at the C-3 position of Xyl*p* residues (Figure 2). In contrast, the signals at δ 5.23 and 5.28 ppm were assigned to Ara*f* (1→3) and Ara*f* (1→2) linkages, representing disubstituted Xyl*p* residues at the C-3 and C-2 positions, respectively. Although the Ara*f* (1→2) monosubstituted linkage is typically detected near δ 5.20 ppm [44], it appeared less frequently than C-3 monosubstitution and was observed only in certain samples. Anomeric signals of unsubstituted and monosubstituted Xyl*p* residues were detected between δ 4.40 and 4.70 ppm. Specifically, the H-1 signal of unsubstituted Xyl*p* was observed at δ 4.49 ppm, whereas the corresponding H-2 signal appeared at δ 3.32 ppm [45,46]. These spectral features confirm the presence of AX in the extracted fractions.

**Figure 3:**
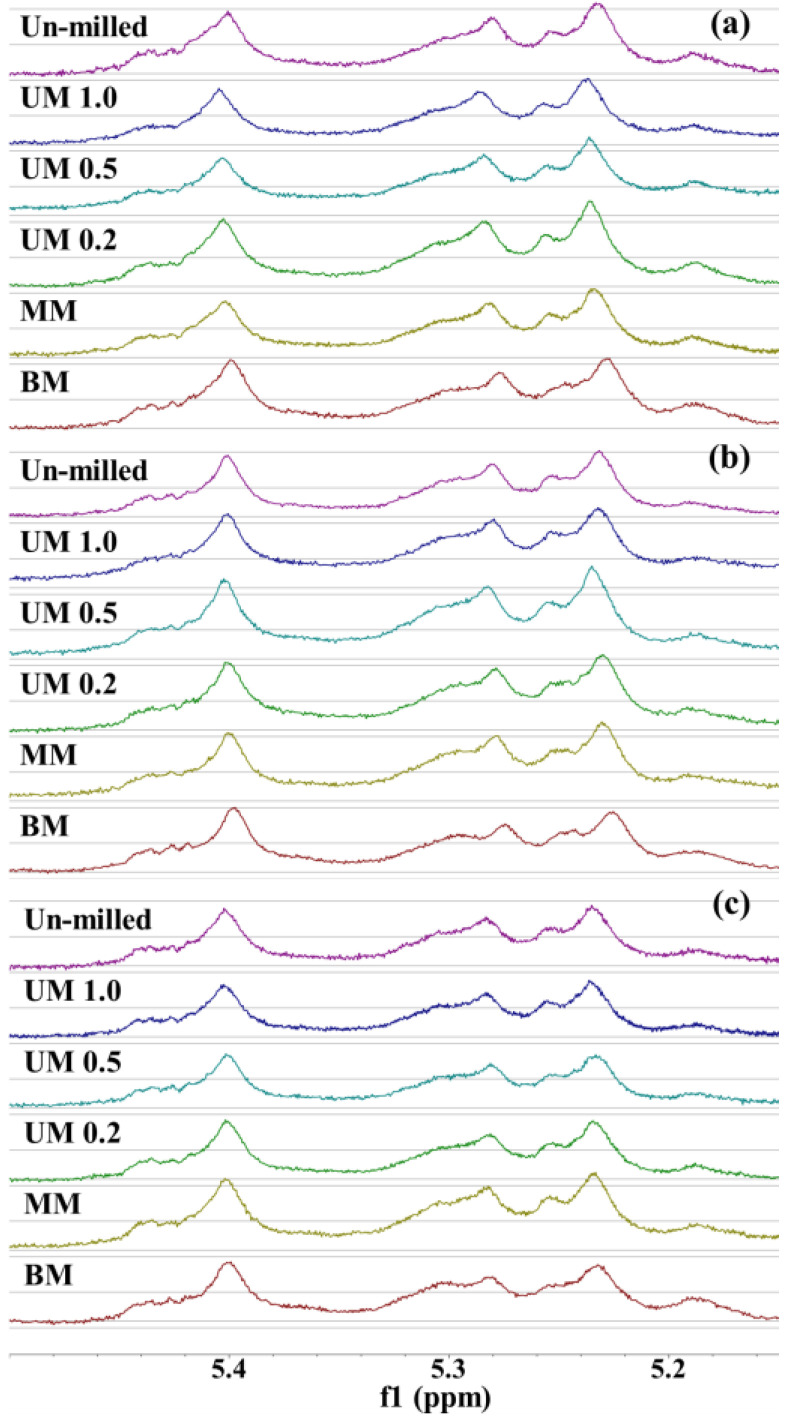
The integration region (δ 5.15–5.50 ppm) from the ^1^H NMR spectra of arabinoxylan (AX) fractions in each variety. (**a**) GS, (**b**) HJ, (**c**) JM.

Based on previous studies [47,48], the proportions of mono-, di-, and unsubstituted Xyl*p* residues and the mono/di (M/D) ratio were calculated by integrating the anomeric proton signals of Ara*f* (δ 5.40, 5.28, and 5.23 ppm). The integration region (δ 5.15–5.50 ppm) is shown in Figure 3. Among the three cultivars, the M/D ratios ranged from 0.95 to 1.16 in GS, 1.01 to 1.15 in HJ, and 1.07 to 1.15 in JM, with generally similar values or slightly higher ratios observed in JM (Table 5). Variations in the degree of substitution of AX have been associated with functional properties, such as solubility, viscosity, and prebiotic potential [48]. A higher M/D ratio reflects fewer arabinose substitutions in AX, indicating a lower degree of substitution.

The strong impact and frictional forces generated during ball milling likely disrupted the disubstituted xylose residues, leading to a relative increase in the monosubstituted structures and a higher M/D ratios. Overall, the different milling treatments did not markedly affect the structural features of AX, with no distinct trend observed between particle size reduction and the degree of xylose substitution. Mechanical forces during milling can disrupt cell wall structures, thereby affecting the A/X ratio and relative proportions of WU-AX and WE-AX [16]. Thus, although changes in the M/D ratio were detected, it remains unclear whether conversion from WU-AX to WE-AX occurred, indicating the need for further investigation.

### 2.6. Monosaccharide Composition of AX

Xylose and arabinose were the major monosaccharide components of the AX fraction in all cultivars (Table 6). The proportion of xylose (56.69–63.14%) was nearly double that of arabinose (26.82–34.63%). However, the monosaccharide composition did not differ significantly between milling treatments. The glucose content ranged from 3.92% to 14.32%, indicating that the amylase reaction was insufficient. This residual glucose likely originates from incomplete starch breakdown during extraction, which occurs through both mechanical disruption and enzymatic hydrolysis. In addition, the lower residual glucose content in AX from the BM bran samples suggests higher purity than that of AX from the other bran samples. In contrast, the higher glucose contents of the HJ-UM samples suggest lower AX purity [49]. Moreover, galactose content was low and stable across milling treatments, whereas xylose content remained consistent despite high variability among samples.

The degree of arabinose branching in AX, represented by the A/X ratio, differed according to cultivar and milling method. This ratio varies among cereal types and varieties and affects the structural and functional properties of AX. Wheat bran-derived AX typically has an A/X ratio of ~0.6, although it varies across the bran layers [50,51]. In this study, the A/X ratios ranged from 0.44 to 0.60. With the exception of GM, UM bran generally decreased the A/X ratio, whereas BM increased it, with values of 0.57 for HJ and 0.60 for JM. This difference may be attributable to wheat-type variation: GM appears more similar to soft wheat and produced a larger proportion of small bran particles (D_10_) than HJ and JM (Table 1) even after UM and MM. However, this finding should be confirmed in future work because the present study included only a limited number of cultivars. In addition, these results suggest that intensive milling may increase the A/X ratio by opening the cell wall network and making more highly substituted A/X domains more accessible and/or solubilizable. The decrease in A/X ratio reported previously for milled samples [13] was observed in WE-AX, which differs from the TAX evaluated in the present study. Although the A/X ratio may influence hydration and swelling behavior, substitution pattern and molecular weight also strongly affect water binding and swelling.

### 2.7. TPC and Antioxidant Activity of AX

Like ferulic acid in AX, TPC is another bioactive compound that contributes to health benefits. The AX fractions from wheat bran showed clear differences in TPC and antioxidant activity, depending on milling technique and particle size reduction (Table 7). Wheat bran contains substantial amounts of ferulic acid, predominantly bound to the cell wall matrix [52]. The feruloylated AX—the major phenolic component of AX—reportedly contains 4.7–10.8 mg FAE/g [53]. In this study, free phenolic acid levels varied among cultivars, ranging from 3.3 to 10.4 mg FAE/g in GS, 5.0 to 11.2 mg FAE/g in HJ, and 4.0 to 18.3 mg FAE/g in JM. Bound phenolic acid concentrations were comparatively lower with narrower ranges: 4.1–6.0 mg FAE/g in GS, 3.9–7.5 mg FAE/g in HJ, and 5.4–6.8 mg FAE/g in JM.

Ball milling consistently yielded higher free phenolic content across all cultivars, confirming that intensive milling facilitates the release of phenolics by disrupting ester linkages between ferulic acid and polysaccharides [54,55]. By contrast, ultracentrifugal (UM 1.0 and UM 0.5) and mortar milling treatments, which retained relatively larger particle sizes, had negligible effects on free phenolic release. These findings underscore the importance of milling intensity in modulating phenolic availability. In contrast, the bound phenolic levels were relatively unaffected by milling, indicating that a considerable fraction of phenolics remains tightly integrated within the cell wall matrix [27].

The antioxidant capacity of the AX fractions, measured using ABTS radical scavenging activity, ranged from 1063 to 2920 mg TE/100 g in GS, 1.494 to 2805 mg TE/100 g in HJ, and 1254 to 2713 mg TE/100 g in JM. BM treatment enhanced the phenolic content and antioxidant capacity; however, these parameters were not strictly proportional. Notably, JM contained the highest TPC under the BM treatment (25.1 mg FAE/g), yet its antioxidant activity (2713 mg TE/100 g) was lower than that of GS (2920 mg TE/100 g). This discrepancy indicates that antioxidant potential is influenced not only by the TPC but also by factors such as cultivar genotype, phenolic structural features, and the positional form of phenolics (free vs. bound). Overall, these results demonstrate that particle size reduction through milling, particularly under BM conditions, enhances the release of phenolics and strengthens the antioxidant capacity of the AX fractions.

### 2.8. In Vitro Fermentation of AX

**Figure 4:**
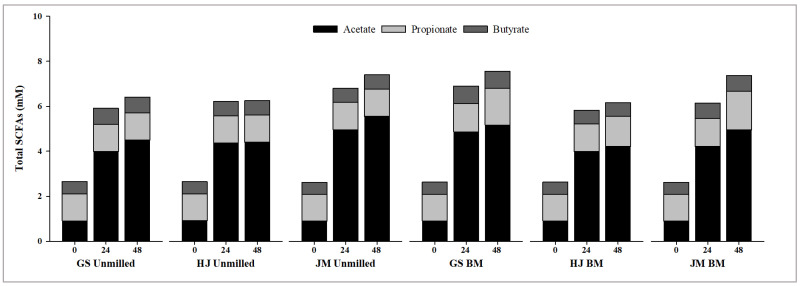
SCFA concentration of arabinoxylan (AX) using un-milled and ball-milled wheat bran during in vitro fermentation of AX for 48 h.

The fermentability of AX extracted from the un-milled and BM bran was assessed over 48 h of in vitro fecal fermentation. Overall, both AXs were readily fermented; however, the extent and pattern of fermentation differed between treatments and cultivars (Figure 4).

Across all samples, acetate was the primary SCFA, followed by propionate and butyrate, consistent with the findings of Feng et al. [56] and Yang et al. [57] for wheat AX. Total SCFA concentrations progressively increased from the starting point (~2.6 mM) to 48 h. Un-milled AX generated total SCFA levels of 6.25–7.40 mM at 48 h, whereas BM bran AX produced values ranging from 6.16 to 7.52 mM, depending on the cultivar. The BM treatment resulted in slightly higher SCFA formation in GS, primarily due to increased acetate. In contrast, HJ and JM showed only minor differences between treatments, suggesting that the impact of the cultivar was minimal. In addition, BM bran AX increased propionate levels at 48 h compared with those of un-milled bran; however, butyrate levels remained relatively low and did not differ substantially between those of un-milled and BM bran AX.

The pH decreased during fermentation, with un-milled AX consistently exhibiting lower pH values than those of BM AX at 24 and 48 h. After 48 h, the un-milled samples reached a pH of 4.36–4.54, whereas the BM samples retained a slightly higher pH (4.35–4.99). This indicates stronger acidification with un-milled AX despite similar or marginally lower SCFA concentrations than those of BM AX. In this study, analyses after digestion in the gastric and intestinal phase were not conducted, these will be worthwhile to evaluate in future work.

Overall, these results demonstrate that, while AXs from wheat bran support microbial fermentation, particle size reduction does not uniformly enhance fermentability. Ball milling improved acetate production in certain cultivars; however, un-milled AX resulted in greater overall fermentation.

## 3. Materials and Methods

### 3.1. Materials

Three wheat cultivars, GS, HJ, and JM, were provided by the National Institute of Crop Science in Korea (Wanju-Gun, Republic of Korea). The wheat bran samples were milled using a Buhler mill (MLU 202, Uzwil, Switzerland). Amyloglucosidase (A7095), bile bovine (B3883), ferulic acid (128708), heat-stable *α*-amylase (A3403), pancreatin (P7545), pepsin (P7012), and protease (P4860) were purchased from Sigma-Aldrich (St. Louis, MO, USA). Endo-*β*-D-glucanase (EC 3.2.1.73) from *Bacillus subtills* was purchased from Megazyme. All chemicals were of analytical grade.

### 3.2. Sample Preparation

Wheat bran samples were ground into smaller particle sizes using three different milling techniques: ultracentrifugal, mortar, and ball (FM 200, MG 200, and BM 200; POWTEQ, Beijing, China). The ultracentrifugal mill (UM) was equipped with a 12-tooth rotor and ring sieves of 1.0, 0.5, and 0.2 mm. A 100 g sample of bran was milled at a rotor speed of 6000 rpm, and the resulting bran fractions were designated as UM 1.0, UM 0.5, and UM 0.2.

For the mortar mill treatment, 50 g of bran was milled under the following conditions: 2 h milling time, 5 min cycle time, 5 min interval time, and 130 rpm. The sample was designated as MM.

For the ball-mill treatment, 21.65 g of bran was placed in a jar containing 200 stainless steel beads (10 mm in diameter), which occupied approximately 35% of the total volume. Milling was performed for 5 h with a cycle time of 10 min, an interval time of 5 min, and a rotational speed of 250 rpm. This sample was designated as the BM.

### 3.3. Measurement of Wheat Bran Particle Size Distribution

The particle size distribution of the bran samples was determined using LS 13 320 laser diffraction particle size analyzer (Beckman Coulter, Brea, CA, USA) equipped with a dry powder dispersion unit and vacuum delivery system. A range of particles with diameters between 0.07 and 2000 μm was obtained.

### 3.4. Wheat Bran Chemical Composition Analysis

The moisture and ash content of bran were determined according to AACC Methods 44-15.02 and 08-01.01, respectively [58]. The total and water-extractable AX (TAX and WE-AX) contents were determined using the phloroglucinol colorimetric method [59]. The total and damaged starch contents were determined using a Total starch assay AA/AMG kit and Starch damage assay kit (Megazyme, Wicklow, Ireland) according to AACC 76-13.01 and 76-31.01, respectively [58].

### 3.5. Isolation and Purification of AX from Wheat Bran

AX was extracted following the method described by Izydorczyk et al. [60] with slight modifications. Wheat bran was mixed with ethanol (1:5, *w*/*v*) and stirred for 1 h to remove lipids. Following filtration using a Büchner funnel and filter paper, the defatted bran was dried at 25 °C for 48 h. Dried bran (35 g) was suspended in 175 mL of distilled water and subjected to sequential enzymatic treatments for destarching and deproteinization.

The suspension was adjusted to pH 6.5, and 66.8 μL of heat-stable α-amylase was added, followed by incubation at 90 °C for 2 h. After cooling, the pH was adjusted to 7.5, and 112.81 μL of protease was added, with incubation at 60 °C for 2 h. The pH was adjusted to 5.5, and 69.1 μL of amyloglucosidase and 15 μL of endo-*β*-D-glucanase were added. The reaction was carried out overnight at 60 °C. The enzymatic activity was terminated by heating the mixture in boiling water for 30 min.

The bran residue was separated, and the liquid fraction was centrifuged at 3000× *g* for 15 min. The supernatant was recombined with the bran residue and extracted with 0.7 M NaOH solution (1 L) at 25 °C for 18 h. After centrifugation, the supernatant was mixed with three volumes of ethanol to precipitate polysaccharides. The precipitate was collected and dialyzed against distilled water (molecular weight cut-off: 12–14 kDa) for 48 h. After several washes with distilled water, purified AX was freeze-dried and weighed to determine the yield.

The protein content of AX was determined according to the AACC Method 46-30.01 [58] using a nitrogen conversion factor of 5.7 (N × 5.7). The ferulic acid content of AX was analyzed following the procedure described by Deng et al. [61].

### 3.6. Analysis of AX Using Nuclear Magnetic Resonance (NMR) Spectroscopy

AX samples (5 mg) were dissolved in 500 μL D2O and incubated at room temperature for 6 h, then lyophilized. This dissolution and freeze-drying step was repeated twice to ensure a complete deuterium exchange. The freeze-dried samples were subsequently dissolved in 600 μL of D2O containing 1 mM TSP [(trimethylsilyl)propionic acid-d4 sodium salt] as an internal reference. The mixture was centrifuged (13,000× *g*, 5 min), and the supernatant was transferred into a 5 mm NMR tube for analysis.

^1^H NMR spectra were recorded at 25 °C on a 600 MHz spectrometer (Agilent, Bruker Biospin, Ettlingen, Germany) with D2O as the internal lock. Each spectrum was acquired using 32 scans and referenced to TSP at 0 ppm. All spectral processing steps—exponential line broadening, phase correction, baseline correction, normalization, and integration—were performed using MestReNova 15.1 (Mestrelab Research SL, Santiago, Spain).

The ratio of mono- to disubstituted xylopyranosyl (Xyl*p*) residues (M/D) was determined by integrating the anomeric proton signals of two arabinofuranosyl (Ara*f*) regions (Area I: δ 5.42 to 5.37, Area II: δ 5.32 to 5.25, δ 5.23 to 5.17). Using monosaccharide composition data, the relative proportions of unsubstituted, monosubstituted, and disubstituted Xyl*p* residues were calculated. Signal assignments and calculations were carried out according to Toole et al. [46] and De Man et al. [47].

### 3.7. Analysis of Monosaccharide Composition of AX

The monosaccharide composition of AX was determined using high-performance anion exchange chromatography with pulsed amperometric detection on a DX-600 system (Dionex, CA, USA) equipped with a CarboPac PA-1 column and an ED50 electrochemical detector (Dionex, Sunnyvale, CA, USA). A 20 μL sample volume was injected and eluted with 0.018 M and 0.2 M sodium hydroxide at a flow rate of 1 mL/min. Reference monosaccharides were prepared at a concentration of 0.005% (*w*/*v*). The content of each monosaccharide was expressed as a percentage of the total monosaccharides analyzed, and the AX content was calculated as 0.88 × (% arabinose + % xylose).

### 3.8. Measurement of AX Antioxidant Activity

Free and bound phenolic compounds from AX were extracted according to the method described by Kaur et al. [27]. The total phenolic content (TPC) was determined using the Folin–Ciocalteu method, as described by Bhinder et al. [62], with minor modifications. Briefly, 200 μL of the extracted sample was diluted with 2.4 mL of distilled water, then 300 μL of Folin–Ciocalteu reagent was added. After 8 min of reaction, 900 μL of sodium carbonate solution (20%, *w*/*v*) was added, and the mixture was incubated at 40 °C for 30 min. The absorbance was measured at 765 nm using a spectrophotometer (X-ma 6100 PC, Human Corporation, Seoul, Republic of Korea). A calibration curve was prepared using trans-ferulic acid, and the results are expressed as milligram ferulic acid equivalents per gram (mg FAE/g) of sample.

The antioxidant activity of free and bound phenolic extracts was evaluated using the ABTS radical scavenging method [63]. In brief, 100 μL of each extract was mixed with 1.85 mL of diluted ABTS reagent and allowed to react for 30 min. The absorbance was recorded at 734 nm, and the antioxidant activity was expressed as milligram Trolox equivalents per 100 g (mg TE/100 g) of sample.

### 3.9. Analysis of In Vitro Fecal Fermentation of AX

In vitro batch digestion was conducted to evaluate the fermentability of AX extracted from BM and un-milled bran with different particle sizes. AX samples were digested using INFOGEST 2.0 protocol [64]. The resulting residues were fermented using human fecal matter obtained from a healthy adult donor with no history of gastrointestinal disorders or antibiotic treatment in the past 3 months. Fecal samples were collected in sterile containers, immediately frozen at −20 °C, and thawed immediately prior to use.

For each sample, 100 mg of digested AX was inoculated with a fecal slurry mixed with medium (10%, *v*/*v*) to a final volume of 50 mL. Fermentation was performed at 37 °C for 48 h, and pH values were recorded at 0, 24, and 48 h. All steps were carried out under anaerobic conditions.

For short-chain fatty acid (SCFA) analysis, the samples were centrifuged at 3000× *g* for 15 min, and the supernatant was collected. Individual SCFAs (acetate, propionate, and butyrate) were quantified using gas chromatography–mass spectrometry (TSQ 9000, Thermo Scientific, Waltham, MA, USA) equipped with a HP-FFAP column (Agilent, Santa Clara, CA, USA). Quantification was performed using external calibration curves prepared using analytical standards.

### 3.10. Statistical Analysis

All data were collected through at least three repeated measurements, and statistical validation of the samples was performed using analysis of variance (ANOVA) with Tukey’s honest significant difference (HSD) test at a significance level of *p* < 0.05, using the SPSS Statistics program (ver. 27.0, SPSS Inc., Armonk, NY, USA).

## 4. Conclusions

Taken together, the results suggest that the milling method and particle size reduction markedly influence the extraction efficiency, structural composition, and antioxidant functionality of AX from wheat bran. Ball milling, which more effectively disrupts the bran cell wall matrix and thereby reduces particle size, consistently outperforms ultracentrifugal and mortar milling treatments in increasing AX yield and releasing free phenolics associated with higher antioxidant capacity. Although the overall substitution patterns (mono-, di-, and unsubstituted xylose) of AX are relatively stable, the BM treatment reduces disubstituted xylose residues while increasing the A/X ratio in some cultivars. These structural changes suggest that intensive mechanical processing alters the polysaccharide structure in ways that favor functional benefits. Overall, the results supported our hypothesis that AX extractability is driven primarily by the degree of bran particle size reduction achieved by different milling techniques, but did not fully support the hypothesis that AX structural characteristics differ hard and soft wheat bran, likely due to a limited number of cultivars evaluated.

Hence, optimizing milling parameters, such as mill type, duration, and particle size, is essential to enhancing the bioactive potential of wheat bran-derived AX in functional foods and applications. Despite the limited number of cultivars tested, the effect of milling appears to be cultivar-dependent. Further research is needed to assess the health-promoting effects of AXs from a broader range of wheat cultivars with diverse structural features, including effects on digestibility, gut microbial fermentation, and in vivo antioxidant activity.

## Figures and Tables

**Table 1:** Particle size distribution of wheat bran.

**Milling** **Treatment ^(1)^**	**D_10_ (μm)**			**D_50_ (μm)**		
	**GS**	**HJ**	**JM**	**GS**	**HJ**	**JM**
Un-milled	542.9 ± 0.0 ^Ad(2)^	284.1 ± 46.8 ^Bc^	429.7 ± 30.7 ^Ac^	1221.3 ± 0.0 ^Cf^	1134.0 ± 11.9 ^Af^	1116.5 ± 16.6 ^Be^
UM 1.0	81.8 ± 9.0 ^Ac^	148.3 ± 33.6 ^Bb^	142.0 ± 11.8 ^Ab^	875.0 ± 9.1 ^Ce^	833.3 ± 12.9 ^Ae^	805.9 ± 8.5 ^Bd^
UM 0.5	15.5 ± 0.2 ^Ab^	19.9 ± 0.4 ^Ba^	35.8 ± 0.1 ^Aa^	353.9 ± 4.9 ^Cc^	343.9 ± 8.0 ^Ac^	378.2 ± 2.7 ^Bc^
UM 0.2	9.8 ± 0.1 ^Aab^	11.9 ± 0.1 ^Ba^	15.3 ± 0.1 ^Aa^	151.2 ± 3.3 ^Cb^	166.7 ± 2.1 ^Ab^	159.5 ± 0.9 ^Bb^
MM	17.3 ± 0.5 ^Ab^	18.9 ± 0.7 ^Ba^	25.7 ± 0.9 ^Aa^	727.1 ± 3.4 ^Cd^	690.9 ± 12.3 ^Ad^	800.9 ± 10.3 ^Bd^
BM	4.1 ± 0.0 ^Aa^	3.0 ± 0.0 ^Ba^	3.0 ± 0.1 ^Aa^	12.3 ± 0.1 ^Ca^	10.0 ± 0.1 ^Aa^	9.8 ± 0.2 ^Ba^
**Milling** **Treatment**	**D_90_ (μm)**			**Span**		
	**GS**	**HJ**	**JM**	**GS**	**HJ**	**JM**
Un-milled	1835.7 ± 0.0 ^Bf^	1794.1 ± 4.0 ^Af^	1778.2 ± 7.4 ^Bf^	1.1 ± 0.0 ^Ba^	1.5 ± 0.2 ^Aa^	1.2 ± 0.0 ^Aa^
UM 1.0	1636.3 ± 2.7 ^Be^	1522.9 ± 36.0 ^Ae^	1584.8 ± 9.5 ^Bd^	1.8 ± 0.0 ^Bb^	1.7 ± 0.1 ^Aa^	1.8 ± 0.0 ^Ab^
UM 0.5	782.4 ± 14.0 ^Bc^	751.2 ± 7.1 ^Ac^	878.8 ± 11.0 ^Bc^	2.2 ± 0.0 ^Bc^	2.1 ± 0.1 ^Ab^	2.2 ± 0.0 ^Ad^
UM 0.2	520.5 ± 6.7 ^Bb^	546.3 ± 7.5 ^Ab^	492.3 ± 2.7 ^Bb^	3.4 ± 0.1 ^Bd^	3.2 ± 0.0 ^Ad^	3.0 ± 0.0 ^Af^
MM	1542.0 ± 6.2 ^Bd^	1463.5 ± 14.6 ^Ad^	1620.9 ± 6.7 ^Be^	2.1 ± 0.0 ^Bc^	2.1 ± 0.0 ^Ab^	2.0 ± 0.0 ^Ac^
BM	45.2 ± 2.0 ^Ba^	29.0 ± 0.2 ^Aa^	30.3 ± 0.6 ^Ba^	3.3 ± 0.2 ^Bd^	2.6 ± 0.0 ^Ac^	2.8 ± 0.1 ^Ae^

^(1)^ UM 1.0, UM 0.5, and UM 0.2: ultracentrifugal mill with 1.0 mm, 0.5 mm, and 0.2 mm sieves, respectively; MM: mortar mill, BM: ball mill. ^(2)^ Results are expressed as mean ± SD. Values with different letters within the same column indicate significant differences (*p* < 0.05) for a given parameter among milling treatments (un-milled, UM, MM, BM) (lowercase letters) or among wheat cultivars (uppercase letters) for the milled bran.

**Table 2:** Chemical composition of wheat bran.

**Milling** **Treatment ^(1)^**	**Moisture (%)**			**Ash (%)**		
	**GS**	**HJ**	**JM**	**GS**	**HJ**	**JM**
Un-milled	12.9 ± 0.0 ^Cf(2)^	12.2 ± 0.2 ^Ac^	11.8 ± 0.0 ^Bd^	4.0 ± 0.0 ^Bab^	3.6 ± 0.0 ^Aa^	5.8 ± 0.0 ^Cbc^
UM 1.0	12.4 ± 0.0 ^Ce^	11.9 ± 0.1 ^Ac^	12.1 ± 0.1 ^Be^	3.9 ± 0.0 ^Ba^	3.7 ± 0.1 ^Aab^	5.7 ± 0.1 ^Ca^
UM 0.5	11.2 ± 0.1 ^Cc^	11.0 ± 0.2 ^Ab^	11.1 ± 0.1 ^Bc^	4.2 ± 0.0 ^Bd^	3.7 ± 0.0 ^Ab^	5.9 ± 0.0 ^Ccd^
UM 0.2	10.2 ± 0.0 ^Ca^	9.8 ± 0.2 ^Aa^	10.3 ± 0.1 ^Ba^	4.1 ± 0.0 ^Bc^	3.8 ± 0.0 ^Ab^	5.9 ± 0.0 ^Cd^
MM	11.4 ± 0.0 ^Cd^	11.1 ± 0.1 ^Ab^	10.8 ± 0.1 ^Bb^	4.1 ± 0.0 ^Bc^	3.8 ± 0.0 ^Ab^	5.8 ± 0.0 ^Cab^
BM	10.4 ± 0.1 ^Cb^	9.9 ± 0.0 ^Aa^	10.7 ± 0.0 ^Bb^	4.0 ± 0.0 ^Bb^	3.7 ± 0.0 ^Aab^	6.0 ± 0.0 ^Cd^
**Milling** **Treatment**	**Damaged Starch (%)**			**Total Starch (%)**		
	**GS**	**HJ**	**JM**	**GS**	**HJ**	**JM**
Un-milled	1.0 ± 0.0 ^Ca^	0.9 ± 0.0 ^Ba^	1.2 ± 0.0 ^Ab^	29.1 ± 0.6 ^Cab^	23.3 ± 0.6 ^Ba^	16.1 ± 0.7 ^Aab^
UM 1.0	1.0 ± 0.0 ^Ca^	1.1 ± 0.0 ^Bb^	1.0 ± 0.0 ^Aa^	30.5 ± 0.0 ^Cb^	24.0 ± 0.2 ^Ba^	18.9 ± 0.5 ^Ac^
UM 0.5	1.4 ± 0.0 ^Cb^	1.5 ± 0.0 ^Bc^	1.1 ± 0.0 ^Ab^	28.4 ± 1.1 ^Ca^	23.7 ± 0.5 ^Ba^	17.4 ± 1.0 ^Abc^
UM 0.2	1.8 ± 0.0 ^Cc^	2.0 ± 0.0 ^Bd^	1.5 ± 0.0 ^Ac^	30.5 ± 0.6 ^Cb^	23.8 ± 0.6 ^Ba^	17.4 ± 0.8 ^Abc^
MM	5.6 ± 0.1 ^Cd^	4.8 ± 0.0 ^Be^	3.6 ± 0.0 ^Ad^	29.2 ± 0.7 ^Cab^	22.2 ± 1.4 ^Ba^	17.3 ± 0.4 ^Abc^
BM	20.7 ± 0.3 ^Cf^	18.8 ± 0.1 ^Bf^	13.7 ± 0.0 ^Ae^	28.1 ± 0.9 ^Ca^	22.6 ± 0.8 ^Ba^	15.2 ± 0.6 ^Aa^

^(1)^ UM 1.0, UM 0.5, and UM 0.2: ultracentrifugal mill with 1.0 mm, 0.5 mm, and 0.2 mm sieves, respectively; MM: mortar mill, BM: ball mill. ^(2)^ Results are expressed as mean ± SD. Values with different letters within the same column indicate significant differences (*p* < 0.05) for a given parameter among milling treatments (un-milled, UM, MM, BM) (lowercase letters) or among wheat cultivars (uppercase letters) for the milled bran.

**Table 3:** Total and water-extractable arabinoxylan content of wheat bran.

Milling Treatment ^(1)^	TAX (%)			WE-AX (%)		
	GS	HJ	JM	GS	HJ	JM
Un-milled	1.87 ± 0.01 ^Aa(2)^	2.35 ± 0.01 ^Aa^	3.76 ± 0.06 ^Aa^	1.22 ± 0.01 ^Ba^	1.25 ± 0.01 ^Ca^	1.17 ± 0.00 ^Aa^
UM 1.0	4.70 ± 0.06 ^Ab^	4.71 ± 0.08 ^Ab^	6.05 ± 0.18 ^Ab^	1.27 ± 0.01 ^Bb^	1.28 ± 0.00 ^Cb^	1.22 ± 0.01 ^Ac^
UM 0.5	8.46 ± 0.19 ^Ac^	8.23 ± 0.03 ^Ac^	7.48 ± 0.07 ^Ac^	1.39 ± 0.00 ^Bc^	1.29 ± 0.00 ^Cb^	1.25 ± 0.00 ^Ad^
UM 0.2	9.19 ± 0.18 ^Ad^	11.12 ± 0.63 ^Ad^	7.65 ± 0.03 ^Ac^	1.37 ± 0.01 ^Bc^	1.36 ± 0.01 ^Cc^	1.26 ± 0.00 ^Ad^
MM	8.50 ± 0.13 ^Ac^	4.69 ± 0.12 ^Ab^	6.96 ± 0.37 ^Ac^	1.25 ± 0.00 ^Bb^	1.24 ± 0.01 ^Ca^	1.20 ± 0.01 ^Ab^
BM	15.36 ± 0.39 ^Ae^	18.26 ± 0.54 ^Ae^	16.82 ± 0.49 ^Ad^	1.79 ± 0.01 ^Bd^	2.02 ± 0.02 ^Cd^	1.75 ± 0.01 ^Ae^

^(1)^ UM 1.0, UM 0.5, and UM 0.2: ultracentrifugal mill with 1.0 mm, 0.5 mm, and 0.2 mm sieves, respectively; MM: mortar mill, BM: ball mill. ^(2)^ Results are expressed as mean ± SD. Values with different letters within the same column indicate significant differences (*p* < 0.05) for a given parameter among milling treatments (un-milled, UM, MM, BM) (lowercase letters) or among wheat cultivars (uppercase letters) for the milled bran.

**Table 4:** Extraction yield and composition of arabinoxylan.

**Milling** **Treatment ^(1)^**	**Defatted Bran (g)**				**Loss Rate (%)**				**AX Yield (g/100g Fraction)**			
	**GS**	**HJ**		**JM**	**GS**	**HJ**		**JM**	**GS**	**HJ**		**JM**
Un-milled	90.8	90.7		92.7	10.1	10.3		7.8	7.4	5.9		4.2
UM 1.0	89.9	89.8		89.9	10.0	11.4		11.2	5.0	4.9		5.0
UM 0.5	90.1	89.3		90.2	11.0	12.0		10.9	6.4	6.2		5.5
UM 0.2	90.3	89.9		90.7	9.6	11.3		10.3	8.1	6.7		4.2
MM	91.5	90.7		92.0	9.3	10.3		8.7	7.8	4.9		4.6
BM	89.6	89.5		91.1	11.6	11.8		9.8	8.9	11.2		6.0
**Milling** **Treatment**	**Protein (%)**						**Ferulic Acid (μg FA/mg)**					
	**GS**		**HJ**		**JM**		**GS**		**HJ**		**JM**	
Un-milled	3.8 ± 0.1 ^Ab(2)^		5.9 ± 0.1 ^Bd^		5.6 ± 0.1 ^Cb^		2.6 ± 0.1 ^Aa^		4.3 ± 0.1 ^Ba^		2.5 ± 0.1 ^Aa^	
UM 1.0	6.2 ± 0.0 ^Af^		4.2 ± 0.0 ^Bb^		7.0 ± 0.1 ^Cd^		2.8 ± 0.0 ^Aa^		4.1 ± 0.1 ^Ba^		2.5 ± 0.1 ^Aa^	
UM 0.5	3.9 ± 0.0 ^Ac^		4.0 ± 0.0 ^Ba^		6.1 ± 0.1 ^Cc^		3.6 ± 0.1 ^Ab^		4.3 ± 0.0 ^Ba^		3.4 ± 0.1 ^Ab^	
UM 0.2	4.1 ± 0.0 ^Ad^		4.2 ± 0.0 ^Bb^		6.2 ± 0.0 ^Cc^		3.6 ± 0.2 ^Ab^		5.4 ± 0.2 ^Bb^		2.7 ± 0.0 ^Aa^	
MM	2.8 ± 0.1 ^Aa^		4.7 ± 0.0 ^Bc^		5.4 ± 0.0 ^Ca^		2.7 ± 0.0 ^Aa^		4.2 ± 0.1 ^Ba^		3.4 ± 0.4 ^Ab^	
BM	5.0 ± 0.0 ^Ae^		6.8 ± 0.1 ^Be^		8.7 ± 0.0 ^Ce^		6.3 ± 0.2 ^Ac^		7.9 ± 0.1 ^Bc^		7.2 ± 0.2 ^Ac^	

^(1)^ UM 1.0, UM 0.5, and UM 0.2: ultracentrifugal mill with 1.0 mm, 0.5 mm, and 0.2 mm sieves, respectively; MM: mortar mill, BM: ball mill. ^(2)^ Results are expressed as mean ± SD. Values with different letters within the same column indicate significant differences (*p* < 0.05) for a given parameter among milling treatments (un-milled, UM, MM, BM) (lowercase letters) or among wheat cultivars (uppercase letters) for AX fractions.

**Table 5:** Ratio of monosubstituted to disubstituted xylose and proportion of unsubstituted (uXyl), monosubstituted (mXyl), disubstituted (dXyl) xylose residues in arabinoxylan fractions.

**Milling** **Treatment ^(1)^**	**mXyl (%)**			**dXyl (%)**		
	**GS**	**HJ**	**JM**	**GS**	**HJ**	**JM**
Un-milled	17.91 ± 0.15 ^Ac(2)^	19.40 ± 0.24 ^Bd^	19.80 ± 0.66 ^Bc^	17.05 ± 0.07 ^Bc^	18.80 ± 0.12 ^Bc^	18.10 ± 0.33 ^Ad^
UM 1.0	16.75 ± 0.44 ^Ab^	18.19 ± 0.36 ^Bc^	19.13 ± 0.37 ^Bc^	17.62 ± 0.22 ^Bd^	16.90 ± 0.18 ^Bb^	17.43 ± 0.18 ^Ac^
UM 0.5	14.35 ± 0.32 ^Aa^	15.11 ± 0.31 ^Ba^	16.56 ± 0.31 ^Bb^	14.82 ± 0.16 ^Ba^	14.95 ± 0.16 ^Ba^	14.72 ± 0.16 ^Aa^
UM 0.2	16.20 ± 0.26 ^Ab^	18.17 ± 0.54 ^Bc^	16.21 ± 0.41 ^Bab^	16.40 ± 0.13 ^Bb^	16.92 ± 0.27 ^Bb^	14.40 ± 0.20 ^Aa^
MM	18.43 ± 0.38 ^Ac^	17.20 ± 0.34 ^Bb^	15.34 ± 0.04 ^Ba^	18.29 ± 0.19 ^Be^	16.90 ± 0.17 ^Bb^	14.33 ± 0.02 ^Aa^
BM	20.55 ± 0.45 ^Ad^	19.74 ± 0.14 ^Bd^	19.32 ± 0.40 ^Bc^	17.72 ± 0.23 ^Bd^	17.13 ± 0.07 ^Bb^	16.84 ± 0.20 ^Ab^
**Milling** **Treatment ^(1)^**	**uXyl (%)**			**Mono/di**		
	**GS**	**HJ**	**JM**	**GS**	**HJ**	**JM**
Un-milled	65.05 ± 0.07 ^Bc^	61.80 ± 0.12 ^Aa^	62.10 ± 0.33 ^Ca^	1.05 ± 0.01 ^Ab^	1.03 ± 0.02 ^Ba^	1.09 ± 0.06 ^Ca^
UM 1.0	65.62 ± 0.22 ^Bd^	64.90 ± 0.18 ^Ac^	63.43 ± 0.18 ^Cb^	0.95 ± 0.04 ^Aa^	1.07 ± 0.03 ^Bab^	1.10 ± 0.03 ^Ca^
UM 0.5	70.82 ± 0.16 ^Bf^	69.95 ± 0.16 ^Ae^	68.72 ± 0.16 ^Cc^	0.97 ± 0.03 ^Aab^	1.01 ± 0.03 ^Ba^	1.13 ± 0.03 ^Ca^
UM 0.2	67.40 ± 0.13 ^Be^	64.92 ± 0.27 ^Ac^	69.40 ± 0.20 ^Cd^	0.99 ± 0.02 ^Aab^	1.08 ± 0.05 ^Bab^	1.13 ± 0.04 ^Ca^
MM	63.29 ± 0.19 ^Bb^	65.90 ± 0.17 ^Ad^	70.33 ± 0.02 ^Ce^	1.01 ± 0.03 ^Aab^	1.02 ± 0.03 ^Ba^	1.07 ± 0.00 ^Ca^
BM	61.72 ± 0.23 ^Ba^	63.13 ± 0.07 ^Ab^	63.84 ± 0.20 ^Cb^	1.16 ± 0.04 ^Ac^	1.15 ± 0.01 ^Bb^	1.15 ± 0.04 ^Ca^

^(1)^ UM 1.0, UM 0.5, and UM 0.2: ultracentrifugal mill with 1.0 mm, 0.5 mm, and 0.2 mm sieves, respectively; MM: mortar mill, BM: ball mill. ^(2)^ Results are expressed as mean ± SD. Values with different letters within the same column indicate significant differences (*p* < 0.05) for a given parameter among milling treatments (un-milled, UM, MM, BM) (lowercase letters) or among wheat cultivars (uppercase letters) for AX fractions.

**Table 6:** Monosaccharide composition of arabinoxylan.

Wheat Variety	Milling Treatment ^(1)^	Monosaccharides (%)				A/X Ratio
		Arabinose	Xylose	Galactose	Glucose	
**GS**	Un-milled	29.36 ± 1.08 ^Aab(2)^	56.69 ± 2.74 ^Ba^	2.17 ± 0.32 ^Aa^	11.77 ± 1.34 ^Ac^	0.52
	UM 1.0	27.86 ± 1.64 ^Aa^	63.14 ± 1.59 ^Bb^	2.55 ± 0.71 ^Aa^	6.45 ± 0.41 ^Aa^	0.44
	UM 0.5	30.29 ± 1.06 ^Aabc^	61.36 ± 1.48 ^Bb^	2.45 ± 0.13 ^Aa^	6.44 ± 0.36 ^Aa^	0.49
	UM 0.2	32.78 ± 0.33 ^Ac^	58.96 ± 0.45 ^Bab^	2.59 ± 0.07 ^Aa^	5.82 ± 0.02 ^Aa^	0.55
	MM	32.05 ± 1.04 ^Abc^	56.82 ± 1.52 ^Ba^	2.59 ± 0.13 ^Aa^	8.55 ± 0.42 ^Ab^	0.56
	BM	31.55 ± 0.82 ^Abc^	60.67 ± 1.19 ^Bab^	2.17 ± 0.32 ^Aa^	5.19 ± 0.32 ^Aa^	0.52
**HJ**	Un-milled	30.88 ± 1.53 ^Abcd^	59.08 ± 2.20 ^Aa^	2.71 ± 0.21 ^Ab^	7.33 ± 0.47 ^Bb^	0.52
	UM 1.0	26.82 ± 0.42 ^Aa^	59.63 ± 0.13 ^Aa^	2.05 ± 0.16 ^Aa^	11.50 ± 0.39 ^Bc^	0.45
	UM 0.5	29.42 ± 0.42 ^Aabc^	57.11 ± 0.57 ^Aa^	2.51 ± 0.05 ^Aab^	10.96 ± 0.38 ^Bc^	0.52
	UM 0.2	27.98 ± 1.40 ^Aab^	55.23 ± 2.52 ^Aa^	2.47 ± 0.37 ^Aab^	14.32 ± 0.97 ^Bd^	0.51
	MM	32.07 ± 1.98 ^Acd^	59.36 ± 2.41 ^Aa^	2.54 ± 0.15 ^Aab^	6.03 ± 0.38 ^Bab^	0.54
	BM	33.22 ± 0.34 ^Ad^	58.42 ± 0.44 ^Aa^	2.84 ± 0.05 ^Ab^	5.51 ± 0.70 ^Ba^	0.57
**JM**	Un-milled	31.47 ± 0.62 ^Abc^	57.49 ± 0.77 ^Ba^	2.69 ± 0.13 ^Ba^	8.36 ± 0.14 ^Ac^	0.54
	UM 1.0	28.21 ± 0.76 ^Aab^	61.00 ± 1.21 ^Ba^	2.68 ± 0.12 ^Ba^	8.12 ± 0.36 ^Ac^	0.46
	UM 0.5	27.82 ± 1.79 ^Aa^	61.40 ± 3.07 ^Ba^	2.71 ± 0.35 ^Ba^	8.08 ± 0.93 ^Ac^	0.45
	UM 0.2	27.58 ± 0.77 ^Aa^	62.48 ± 1.29 ^Ba^	2.77 ± 0.10 ^Bab^	7.17 ± 0.42 ^Abc^	0.44
	MM	31.77 ± 0.57 ^Ac^	59.15 ± 0.77 ^Ba^	2.71 ± 0.06 ^Ba^	6.36 ± 0.19 ^Ab^	0.53
	BM	34.63 ± 2.09 ^Ac^	58.19 ± 2.88 ^Ba^	3.26 ± 0.24 ^Bb^	3.92 ± 0.62 ^Aa^	0.60

^(1)^ UM 1.0, UM 0.5, and UM 0.2: ultracentrifugal mill with 1.0 mm, 0.5 mm, and 0.2 mm sieves, respectively; MM: mortar mill, BM: ball mill. ^(2)^ Results are expressed as mean ± SD. Values with different letters within the same column indicate significant differences (*p* < 0.05) for a given parameter among milling treatments (un-milled, UM, MM, BM) (lowercases) or among wheat cultivars (uppercase letters) for AX fractions.

**Table 7:** Total phenolic content and antioxidant activity of arabinoxylan.

**Milling** **Treatment ^(1)^**	**Total Phenolic Content (mg FAE/g)**								
	**Free**			**Bound**			**Total**		
	**GS**	**HJ**	**JM**	**GS**	**HJ**	**JM**	**GS**	**HJ**	**JM**
Un-milled	5.2 ± 0.0 ^Ac(2)^	5.0 ± 0.0 ^Ba^	4.3 ± 0.4 ^Cab^	5.7 ± 0.1 ^Ad^	5.1 ± 0.3 ^Ad^	6.0 ± 0.1 ^Bcd^	11.0 ± 0.1 ^Ac^	10.1 ± 0.3 ^Bab^	10.3 ± 0.3 ^Cab^
UM 1.0	3.3 ± 0.1 ^Aa^	5.2 ± 0.1 ^Ba^	4.0 ± 0.0 ^Ca^	5.0 ± 0.3 ^Ac^	5.0 ± 0.4 ^Acd^	6.1 ± 0.2 ^Bd^	8.2 ± 0.4 ^Aa^	10.2 ± 0.3 ^Bb^	10.1 ± 0.2 ^Ca^
UM 0.5	3.9 ± 0.0 ^Ab^	5.1 ± 0.0 ^Ba^	5.4 ± 0.0 ^Cc^	4.4 ± 0.2 ^Aab^	4.5 ± 0.3 ^Abc^	5.4 ± 0.1 ^Ba^	8.2 ± 0.2 ^Aa^	9.6 ± 0.2 ^Bab^	10.8 ± 0.1 ^Cb^
UM 0.2	5.3 ± 0.1 ^Ac^	6.9 ± 0.1 ^Bc^	4.9 ± 0.0 ^Cbc^	4.1 ± 0.2 ^Aa^	4.4 ± 0.1 ^Aab^	5.6 ± 0.1 ^Bab^	9.4 ± 0.2 ^Ab^	11.4 ± 0.2 ^Bc^	10.5 ± 0.1 ^Cab^
MM	4.1 ± 0.3 ^Ab^	5.6 ± 0.1 ^Bb^	4.2 ± 0.0 ^Ca^	4.6 ± 0.2 ^Abc^	3.9 ± 0.2 ^Aa^	5.8 ± 0.1 ^Bbc^	8.7 ± 0.5 ^Aa^	9.5 ± 0.2 ^Ba^	10.0 ± 0.1 ^Ca^
BM	10.4 ± 0.0 ^Ad^	11.2 ± 0.3 ^Bd^	18.3 ± 0.6 ^Cd^	6.0 ± 0.1 ^Ad^	7.5 ± 0.1 ^Ae^	6.8 ± 0.0 ^Be^	16.4 ± 0.0 ^Ad^	18.7 ± 0.4 ^Bd^	25.1 ± 0.6 ^Cc^
**Milling** **Treatment ^(1)^**	**Antioxidant Activity (mg TE/100g)**								
	**Free**			**Bound**			**Total**		
	**GS**	**HJ**	**JM**	**GS**	**HJ**	**JM**	**GS**	**HJ**	**JM**
Un-milled	1657 ± 33 ^Be^	1079 ± 14 ^Cb^	649 ± 16 ^Aa^	604 ± 14 ^Bb^	626 ± 57 ^Bab^	604 ± 23 ^Ac^	2261 ± 31 ^Bf^	1705 ± 69 ^Cb^	1254 ± 26 ^Aa^
UM 1.0	576 ± 7 ^Ba^	918 ± 16 ^Ca^	669 ± 5 ^Aa^	486 ± 59 ^Ba^	600 ± 31 ^Ba^	587 ± 23 ^Ac^	1063 ± 60 ^Ba^	1518 ± 44 ^Ca^	1256 ± 22 ^Aa^
UM 0.5	724 ± 5 ^Bb^	894 ± 30 ^Ca^	1022 ± 32 ^Ac^	616 ± 29 ^Bb^	600 ± 15 ^Bab^	486 ± 54 ^Aa^	1340 ± 32 ^Bb^	1494 ± 31 ^Ca^	1508 ± 75 ^Ac^
UM 0.2	1053 ± 24 ^Bc^	1336 ± 28 ^Cc^	888 ± 6 ^Ab^	545 ± 53 ^Bab^	672 ± 33 ^Bb^	510 ± 28 ^Aa^	1598 ± 76 ^Bc^	2008 ± 22 ^Cc^	1398 ± 32 ^Ab^
MM	1291 ± 54 ^Bd^	1114 ± 8 ^Cb^	679 ± 17 ^Aa^	512 ± 32 ^Ba^	610 ± 17 ^Bab^	585 ± 13 ^Abc^	1804 ± 79 ^Bd^	1723 ± 23 ^Cb^	1264 ± 29 ^Aa^
BM	2122 ± 4 ^Bf^	2160 ± 32 ^Cd^	2138 ± 27 ^Ad^	797 ± 20 ^Bc^	645 ± 21 ^Bab^	575 ± 21 ^Ac^	2920 ± 24 ^Be^	2805 ± 49 ^Cd^	2713 ± 38 ^Ad^

^(1)^ UM 1.0, UM 0.5, and UM 0.2: ultracentrifugal mill with 1.0 mm, 0.5 mm, and 0.2 mm sieves, respectively; MM: mortar mill, BM: ball mill. ^(2)^ Results are expressed as mean ± SD. Values with different letters within the same column indicate significant differences (*p* < 0.05) for a given parameter among milling treatments (un-milled, UM, MM, BM) (lowercase letters) or among wheat cultivars (uppercase letters) for AX fractions.

## Data Availability

The data presented in this study are available upon request from the corresponding author.

## Abbreviations

The following abbreviations are used in this manuscript:

AX	Arabinoxylan
TAX	Total arabinoxylan
WE-AX	Water-extractable arabinoxylan
WU-AX	Water-unextractable arabinoxylan
A/X	Arabinose-to-xylose ratio
GS	Goso
HJ	Hojoong
JM	Joongmo
Araf	Arabinofuranosyl
Xylp	Xylopyranosyl
ABTS	2,2′-Azino-bis-(3-ethylbenzothiazoline-6-sulfonic acid)
TPC	Total phenolic content
SCFA	Short-chain fatty acid
